# Pure laparoscopic bilateral arcuate line hernia repair (with video)

**DOI:** 10.1093/jscr/rjae347

**Published:** 2024-08-08

**Authors:** J Rappoport, J Carrasco, B Gamez

**Affiliations:** Department of Surgery, Hospital Clínico Universidad de Chile, Dr. Carlos Lorca Tobar 999, Independencia, Santiago 83800456, Chile; Department of Surgery, Hospital Clínico Universidad de Chile, Dr. Carlos Lorca Tobar 999, Independencia, Santiago 83800456, Chile; Department of Surgery, Hospital Clínico Universidad de Chile, Dr. Carlos Lorca Tobar 999, Independencia, Santiago 83800456, Chile

**Keywords:** hernia, arcuate line, mesh repair, abdominal wall

## Abstract

An arcuate line hernia is a generally asymptomatic, ascending protrusion of intraperitoneal structures over the linea arcuata. Arcuate line herniae are scarcely reported in the literature. Only a few publications were found. No clear descriptions of the techniques for repair have been published either. We aim to provide diagnostic images and illustrate our method to repair this hernia.

## Introduction

Arcuate line hernia (ALH) is a rare pathology characterized by herniation of the peritoneum ascending from the arcuate line, passing between the rectus abdominis muscle and the aponeurotic fascia that covers it posteriorly. The true prevalence of ALH is unknown. However, it is thought that this is an underdiagnosed pathology in part because it presents mainly asymptomatically [1].

It has been reported in isolation and bilaterally or in association with other abdominal wall hernias. ALH complication has been reported due to strangulation of intestinal loops. Association with increased BMI, diabetes mellitus, and aortic aneurysm has been reported in patients with symptomatic AHL.

The diagnosis of this pathology is exceptional. Its knowledge and dissemination are essential, given the possible surgical complications that derive from this pathology.

## Case report

The patient was a 49-year-old healthy female. She presented with a right-sided paramedian abdominal bulge at the level of the umbilicus. She noticed this swelling 4 years ago because of recurrent abdominal pain in the area. There have been no complicating episodes requiring immediate surgical resolution. On examination, we found a paramedian swelling in the right abdominal wall, which increased during the Valsalva manoeuvre.

A computed tomography (CT) was performed and revealed the presence of a small-bowel loop between the posterior surface of the right rectus muscle and the peritoneum over the ALH, configuring the presence of type III ALH [2]. Surgery was performed with laparoscopic technique. Pneumoperitoneum at 15 mm Hg with a Veress needle at the left Palmer point, one puncture without incident. T1, 10 mm left flank, optiview technique, camera is inserted checking Veress needle in good position without incidental injuries. T2, 5 mm in the left upper quadrant, T3, 10 mm in the left iliac fossa, T4, 5 mm in the left lower quadrant. All under direct vision without incident. At initial laparoscopic exploration, a five cm wide defect was observed in the posterior surface of the right abdominal rectus muscle, constituting a defect with the appearance of a peritoneum pocket in the abdominal wall. The same defect was found at the left side. We also found a large amount of fat covering the midline in the umbilical area, without the presence of umbilical hernia. The defects were closed using v-lock 3–0 plus with continue stiches. An intraperitoneal underlay mesh (Symbotex®, Medtronic) of 10 x 15 cm was placed over the defect and fixated with an absorbable fixation system (Absorba Tack ®, Medtronic).

Total operative time was 150 minutes. The estimated blood loss was less than 10 ml. After an uneventful postoperative course, patient was discharged the next day without complications.

## Discussion

An ALH is a rare pathology, whose clinical presentation and pathophysiology have not been clarified, and there are currently no management or follow-up recommendations. The ubication of the arcuate line (AL) is highly variable. Monkhouse [3] in a study carried out on cadavers, on anatomical variations of the abdominal wall, described that the apex of the AL was on some occasions as high as the umbilicus and on others almost at the level of the pubis, thus forming little more than a foramen for the passage of the inferior epigastric vessels. In addition, in his study he found that the medial end is usually lower than the lateral one, concluding that a symmetrical disposition of the AL is a rarity. We believe that these anatomical variations undoubtedly affect the clinical presentation of this pathology, since they contribute to the weakness of the abdominal wall in different areas.

Regarding the classification proposed by Coulier [2] into three types, it allows to standardize the diagnosis and with it also the treatment scheme. Patients with ALH type 1 do not require surgical treatment, however patients with HLA type 2, and especially type 3, can be symptomatic and even present with acute complications, so surgical treatment is essential.

A review of literature made by Bloemen [1] makes it clear that there is a significant percentage of patients who consult the emergency department and leave without a diagnosis presented ALH as a pathology that could explain their pain. This at least obliges us to consider this diagnosis within the differential diagnosis of a patient with predisposing factors for hernias and who presents abdominal pain in whom the usual studies do not show a defined etiology. The CT analysis is especially important in these cases and both the radiologist, and the clinician should suspect this entity and carefully review the images, since the abdominal wall is not usually considered as an etiology of the pathology.

The proposed therapeutic alternatives range from open, laparoscopic [4], or robotic surgery, including mixed techniques. There seems to be a certain consensus that laparoscopic techniques offer advantages over open ones, by maintaining the integrity of the wall, and allowing greater diagnostic accuracy, unilaterally or bilaterally, as well as evaluating the presence of other abdominal wall hernias.

Further study of the pathology is necessary to determine management and follow-up recommendations.

## Supplementary Material

HERNIA_LINEA_ARCUATA_rjae347
